# Divergent Selection Promotes Intraspecific Genomic Differentiation in *Spodoptera littoralis* With Possible Involvement in Detoxification

**DOI:** 10.1002/ece3.70917

**Published:** 2025-03-17

**Authors:** Karine Durand, Anne‐Laure Clamens, Bruno Le Ru, Youssef Dewer, Frédérique Hilliou, Camille Meslin, Nicolas Nègre, Gael J. Kergoat, Emmanuelle Jacquin‐Joly, Kiwoong Nam

**Affiliations:** ^1^ DGIMI, INRAE Univ Montpellier Montpellier France; ^2^ CBGP, INRAE, CIRAD, IRD, Institut Agro Univ Montpellier Montferrier‐sur‐Lez France; ^3^ Laboratoire Evolution Génomes Comportement et Ecologie, UMR CNRS 9191, IRD 247, Université Paris Sud Université Paris‐ Saclay Gif‐sur‐Yvette France; ^4^ Unité de Recherche UMR 247 African Insect Science for Food and Health (ICIPE) Nairobi Kenya; ^5^ Phytotoxicity Research Department, Central Agricultural Pesticide Laboratory Agricultural Research Center Giza Egypt; ^6^ INRAE, Institut Sophia Agrobiotech Université Côte D'Azur Nice France; ^7^ Sorbonne Université, INRAE, CNRS, IRD, UPEC Université Paris Cité, Institute of Ecology and Environmental Sciences of Paris Versailles France

**Keywords:** cotton leafworm, cytochrome P450, divergent selection, speciation, *Spodoptera littoralis*

## Abstract

The cotton leafworm, *Spodoptera littoralis* (Lepidoptera: Noctuidae), is a major agricultural pest affecting crops like cotton, maize, tomatoes, and wheat across southern Europe, Africa, the Middle East, and western Asia. Whole genome analyses have revealed adaptive evolution in chemosensation and detoxification genes in *S. littoralis* . However, the extent of intraspecific diversity influenced by recent adaptive evolutionary forces remains unclear. In this study, we conducted a population genomics analysis using 31 *S. littoralis* individuals from sub‐Saharan Africa, northern Africa, and southern Europe to assess the existence of intraspecific population divergence and identify the underlying evolutionary forces. We show whole genome differentiation between populations based on geographic origin from the analyzed samples. Phylogenetic analyses indicate that sub‐Saharan and southern European populations share a common ancestor, distinct from several northern African populations. F_ST_ and d_XY_ statistics along the chromosomes reveal loci with restricted gene flow among populations. These loci are associated with population‐specific selective sweeps, indicating the role of divergent natural selection in limiting gene flow. Notably, these loci are enriched with detoxification genes, including cytochrome P450, multidrug resistance, and xanthine dehydrogenase genes, all of which are potentially associated with detoxification. These results demonstrate that divergent selection limits gene flow among geographically distinct populations with the possibility of the involvement of detoxification as a key trait. We argue that this genetic heterogeneity can be considered in pest monitoring and management, as strategies tailored to specific populations may not be relevant for others.

## Graphical abstract

Population genomics analysis of *Spodoptera littoralis* reveals whole genome differentiation based on geographic sampling in Egypt, sub‐Saharan Africa, and Southern Europe. We showed that this differentiation is driven by divergent selection on genes potentially involved in detoxification.

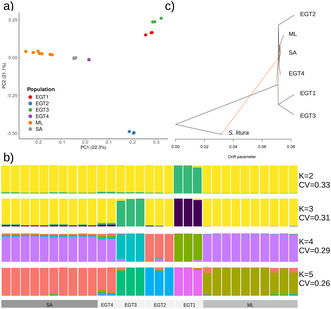

## 1 Introduction

A species deviates from panmixia in the presence of restrictions in gene flow. Allopatry is one of the major factors leading to diversification (Futuyma and Mayer 1980; Mayr 1963; Tishechkin 2020) through geographical distance or natural barriers impeding gene flow, potentially coupled with adaptive evolution, genetic incompatibilities, genetic drift, or founder effects (Barraclough and Vogler 2000; Coyne, Coyne, and Orr 2004; Darwell, Fox, and Althoff 2014; Loretán et al. 2020; Wright 1943). The divergence may also occur among sympatric populations through divergent selection, wherein the strength is sufficiently strong to suppress the effect of gene flow (Bolnick and Fitzpatrick 2007; Feder et al. 2012). The divergence between allopatric or sympatric populations can also be promoted by chromosomal rearrangements and assortative matings (Mackintosh et al. 2023; Rieseberg 2001). Since population divergence is a source of incipient speciation, evolutionary forces underlying the divergence can be a fuel for species diversity.

Population genomics approaches have been increasingly used to investigate the evolutionary history of divergence among populations because whole genome sequences provide ample information on evolutionary processes, such as mutation, natural selection, migration, and demographic changes. Analyses of these genomic data enable us to reconstruct the evolutionary history of populations through bottom‐up approaches without predetermined traits of interest. For example, genomic regions resistant to gene flow can be identified through genome‐wide scans (e.g., F_ST_ , d_XY_ ) (Cruickshank and Hahn 2014). In addition, the footprints of selective sweeps can be detected to test the presence of divergent selection. The function of genes identified from these approaches has been used to infer the evolutionary forces driving genetic differentiation or divergent selection although cause‐and‐effect relationships are yet to be verified (Stankowski et al. 2023).

The cotton leafworm *Spodoptera littoralis* (Boisduval) (Lepidoptera: Noctuidae) is a significant agricultural pest species distributed across southern Europe, Africa, the Middle East, and Western Asia (Pogue 2002). It is a polyphagous species recorded on 130 host plants, including cotton, maize, sugar cane, tomatoes, and wheat, belonging to 56 families, leading to significant economic losses (Pogue 2002). The cotton leafworm has been classified as an A2 quarantine species by the European and Mediterranean Plant Protection Organization since 1981, underscoring its recognition as a considerable threat (EPPO 1981). *S. littoralis* is consistently ranked among the top most insecticide‐resistant arthropod species worldwide (Moustafa et al. 2023; Zhu et al. 2016). The extensive application of insecticides has led to the emergence of resistance across nearly all insecticide groups (Fouad, Ahmed, and Moustafa 2022; Moustafa et al. 2023). *S. littoralis* is expected to extend its range worldwide, which could have a major impact on important crops (ElShahed et al. 2023), highlighting the importance of better understanding its adaptive capacity.

Recent studies based on whole genome data revealed adaptive evolutionary history reshaping the genomic architecture of *S. littoralis* (Meslin et al. 2022; Wu et al. 2022). The genome project of *S. littoralis* revealed dramatically increased copy numbers of gustatory receptors (GR) compared with monophagous and oligophagous lepidopteran species such as *Bombyx mori* L. and *Heliconius melpomene* (L.) (Meslin et al. 2022), as already observed in other polyphagous species such as *S. frugiperda* (J.E. Smith), *S. litura* (Fabricius) and *Helicoverpa armigera* (Hübner) (Cheng et al. 2017; Gouin et al. 2017; Pearce et al. 2017). Interestingly, positive selection was observed from the bitter receptor clade in the GR gene family of *S. littoralis* , indicating selective pressure related to the detection or avoidance of harmful compounds (Meslin et al. 2022). A significant expansion of detoxification gene families has also been reported in *S. littoralis* , with the detection of a higher number of cytochrome P450 (CYP) genes than in *B. mori, Danaus plexippus* (L.), and interestingly, than in the polyphagous pests *S. exigua* (Hübner) and *H. armigera* (Calla et al. 2017; Cheng et al. 2017) that belong to the same subfamily (Noctuinae). The increase in the P450 gene copy number is particularly pronounced in clans 3 and 4, which are well‐known to play a major role in the detoxification of plant secondary metabolites or insecticides (Hilliou et al. 2021; Wu et al. 2022). These findings suggest the adaptive role of chemosensation and detoxification mechanisms in *S. littoralis*.

While these genomic studies have significantly advanced our comprehension of the long‐term evolution that has reshaped genome structure in *S. littoralis* , intraspecific diversity patterns arising from recent evolutionary forces remain unexplored. For example, different geographic populations may be under different strengths of selective pressure, possibly concerning chemosensation or detoxification. Alternatively, population differentiation could be explained solely by geographic separation. Considering the remarkable dispersal ability with probable high levels of gene flow in *S. littoralis* (EFSA PLH Panel 2015; Salama and Shoukry 1972), panmixia could also be observed. To the best of our knowledge, population genomics analyses have not yet been carried out on *S. littoralis*. Thus, we still have a limited understanding of the genetic differentiations promoted by adaptive evolutionary forces, reproductive barriers, and geographic distances in this species.

In this study, we performed population genomics analyses using 31 samples from three different geographic areas including northern Africa (represented by Egypt, EGT), sub‐Saharan Africa (represented by four sub‐Saharan countries, SA), and southern Europe (represented by specimens collected in France, in the Montpellier area, ML) to test the existence of genomic divergence among the populations and to identify underlying evolutionary forces including allopatry and local adaptive evolution responsible for the divergence. It should be noted that we do not aim to reveal the general pattern of *S. littoralis* divergences, nor to describe a local population.

## 2 Materials and Methods

### 2.1 Sampling

**FIGURE 1:**
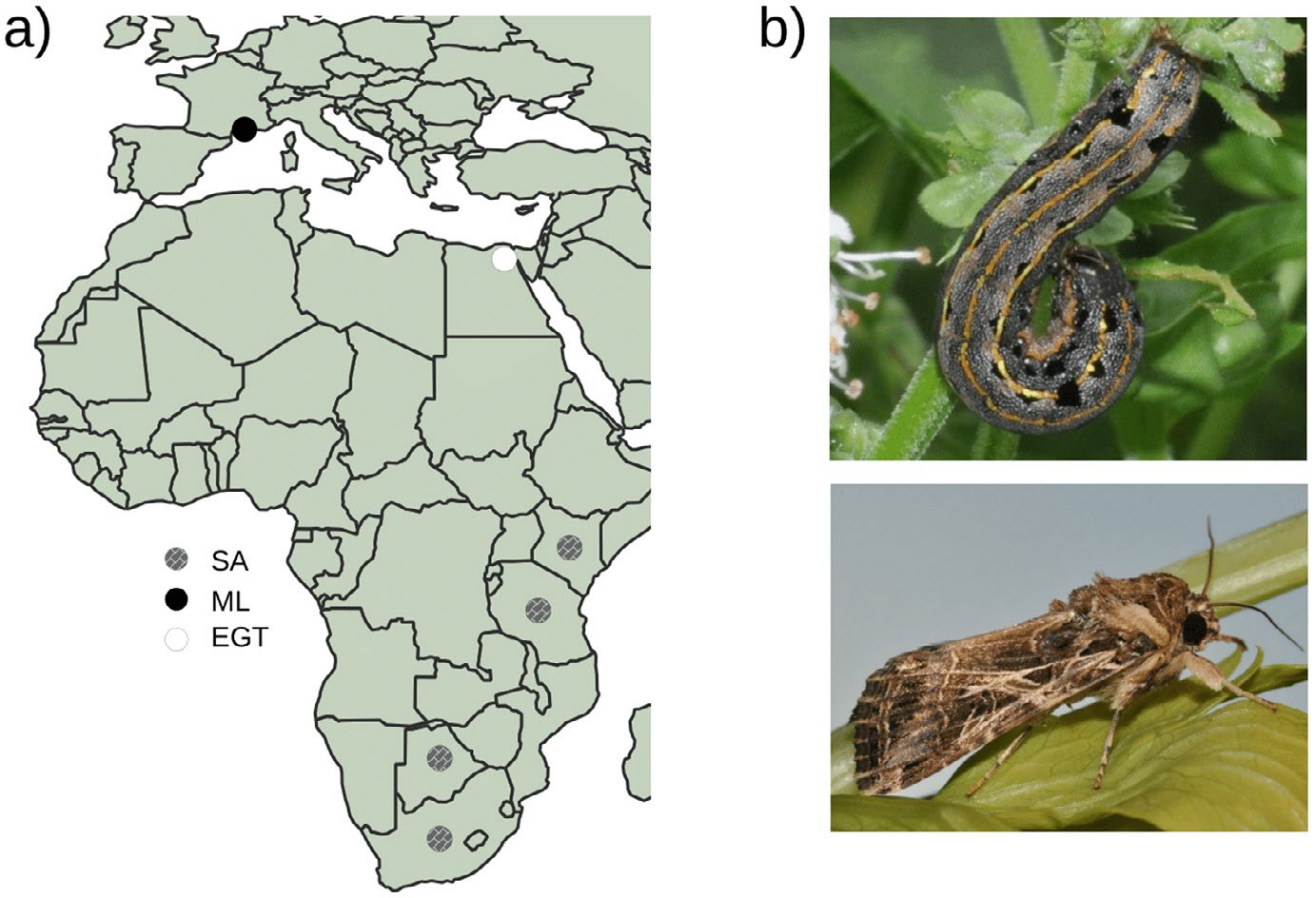
Sampling of *Spodoptera littoralis* samples. (a) Geographic distribution of the 31 *S*. *littoralis* samples used in this study. The map was generated using Natural Earth with QGIS v3.34.3 (QGIS 2024). (b) Photos of *S*. *littoralis* at the larval (top) and adult (bottom) stages.

Samples from EGT were collected from three different localities. Eggs and larvae were collected in Kafr‐El‐Sheikh on cotton and maize, in Faiyum on cotton, and in Damanhour on cotton and tomatoes. Larvae were reared in the laboratory on their respective host plants for one to two weeks until they reached the fifth instar larval stage. Samples from SA were collected with light traps (for Botswana, Kenya, the Republic of South Africa, and Tanzania). Samples from ML were collected in a pheromone trap installed on the campus of the University of Montpellier in France. In total, we analyzed 11 samples from EGT, 10 from SA, and 10 from ML, totaling 31 samples (Figure 1 and Table 1).

**TABLE 1:** The list of the samples used in this study.

ID	Sampling sites		Sampling time	Sampling method and stages	Host plant
	Country	City			
ML10	France	Montpellier	Sep—2018	Light trapping (adults)	Unknown
ML11					
ML13					
ML2					
ML3					
ML4					
ML5					
ML7					
ML8					
ML9					
KNAM_0001	Botswana	Okavango	2016	Light trapping (adult)	Unknown
KNAM_0002					
KNAM_0003					
KNAM_0005	RSA	W. Cape	2016	Light trapping (adult)	Unknown
KNAM_0010	Kenya	Watamu Ruiru Ruiru Ruiru	2016	Light trapping (adult)	Unknown
KNAM_0012					
KNAM_0014					
KNAM_0015					
KNAM_0020	Tanzania	Iboya	2016	Light trapping (adult)	Unknown
KNAM_0021					
KNAM_0049	Egypt	Kafr‐El‐Sheikh	2013	Hand pick (larvae)	Cotton, maize
KNAM_0050					
KNAM_0051					
KNAM_0052					
KNAM_0053	Egypt	Faiyum	2013	Hand pick (larvae)	Cotton
KNAM_0054					
KNAM_0055					
KNAM_0056					
KNAM_0057	Egypt	Damanhour	2013	Hand pick (larvae)	Cotton, tomatoes
KNAM_0059					
KNAM_0060					

### 2.2 Variant Calling

Genomic DNA was extracted using the Promega Wizard Genomic DNA Kit (Promega, Madison, USA). Subsequently, libraries for whole genome resequencing were generated from 1.0 μg of DNA per sample using the NEBNext DNA Library Prep Kit. Paired‐end resequencing was performed using Novaseq S6000 (Illumina, San Diego, CA, USA). The read length was 150 bp, and the read depth was approximately 20X for each sample. Adapter sequences were eliminated from the reads using AdapterRemoval v2.1.7 (Schubert, Lindgreen, and Orlando 2016). The reads were then aligned using bowtie2 v2.3.4.1 (Langmead and Salzberg 2012) with the ‐very‐sensitive‐local preset against the reference genome of *S. littoralis* (GCA902850265) collected in Egypt in 1963, comprising 31 chromosomes with a total size of 435.6 Mb. Subsequently, we performed haplotype calling using GATK v4.1.2.074 (McKenna et al. 2010), followed by merging the resulting gvcf files into a single gvcf file and calling genetic variations. Only single nucleotide variations (SNVs) were retained, and filtering was applied by excluding SNVs with the following criteria: QD less than 2.0, FS exceeded 60.0, MQ dropped below 40.0, MQRankSum was below −12.5, or readPosRankSum below −8.0.

### 2.3 Phylogenetic Analyses Among Populations

We performed a principal component analysis to identify genomically differentiated groups using PLINK v1.9 (Rentería, Cortes, and Medland 2013). The ancestry coefficient analysis was performed to test different shared ancestry among the identified groups using sNMF v1.2 (Frichot et al. 2014) with varying values of *K*, ranging from zero to five. Lastly, a TreeMix v1.13 analysis (Pickrell and Pritchard 2012) was conducted to generate a phylogenetic tree with the identified group. For this task, we generated an additional vcf file by further including 10 outgroup individuals of *S. litura* (SRR5132398, SRR5132410, SRR5132416, SRR5132421, SRR5132425, SRR5132428, SRR5132430, SRR5132440, SRR5132444, and SRR5132445), which was diverged from *S. littoralis* lineage 4.45 million years ago (Kergoat et al. 2021), to the vcf files from *S. littoralis* . *S. littoralis* generally does not have overlapped habitat ranges with *S. litura*, which is found in Asia and Oceania. SNVs with missing genotypes from at least one sample were removed using Vcftools v0.1.13 (Danecek et al. 2011). The vcf file was converted to a TreeMix input file using Stacks (Catchen et al. 2013). Then, the binary of TreeMix was executed with varying numbers of migration events ranging from zero to five, with ‐k 500 to account for linkage disequilibrium, and rooted with *S. litura*.

### 2.4 Divergent Selection Limiting Gene Flow

The loci with restricted gene flow were first identified from Weir and Cockerham's F_ST_ (Weir and Cockerham 1984) using Vcftools v0.1.13, employing a window size of 500 kbp, which corresponds to approximately 0.125% of the whole genome. Outliers of genetic differentiation (OGD) were identified from the top 2% of the F_ST_ . Since the outliers of F_ST_ could be solely generated by reduced intra‐population genetic diversity rather than increased genetic differentiation between populations (Meirmans and Hedrick 2011), d_XY_ was also calculated using Dxy_calculate (https://github.com/hugang123). The same threshold was applied to identify outliers of d_XY_ . Since d_XY_ outliers might be generated due to a local increase in diversity (Irwin et al. 2016), we calculated nucleotide diversity (*π*) using Vcftools v0.1.13 with 100kbp windows to test that the d_XY_ outliers have particularly increased genetic diversity. If a locus exhibits both F_ST_ and d_XY_ outliers, we consider this locus OGD (Outliers of Genetic Differentiation).

We further investigated if divergent natural selection is responsible for the generation of the OGDs by reducing gene flow at selectively targeted loci. We used SweeD v4.0.0 (Pavlidis et al. 2013) to identify loci under selective sweep using a grid of 1,000. We identified outliers of the composite likelihood of selective sweeps ranking in the top 0.05%. If an outlier includes a minimum of two consecutive grids, we consider that this locus was targeted by selective sweep. The role of selective sweeps in genetic differentiation was tested from a non‐random association between OGDs and targets of selective sweeps using a permutation test with 1,000 replications. We determined the function of genes and associated gene ontology (GO) terms by aligning the coding sequences from GCA902850265 to the *S. littoralis* OGS3 proteins (Meslin et al. 2022) using BLAST2GO v6.0.3 (Conesa and Götz 2008) with default parameters. If a OGS3 includes CYP genes, the formal gene names approved by CYP communities were obtained by blasting the genes against CYP genes in OGS2.0 (Meslin et al. 2022). We identified overrepresented GO terms within the selection target within OGD using Fisher's exact test. A statistical significance threshold of False Discovery Rate < 0.1 was applied.

## 3 Results

### 3.1 Population Structure

**FIGURE 2:**
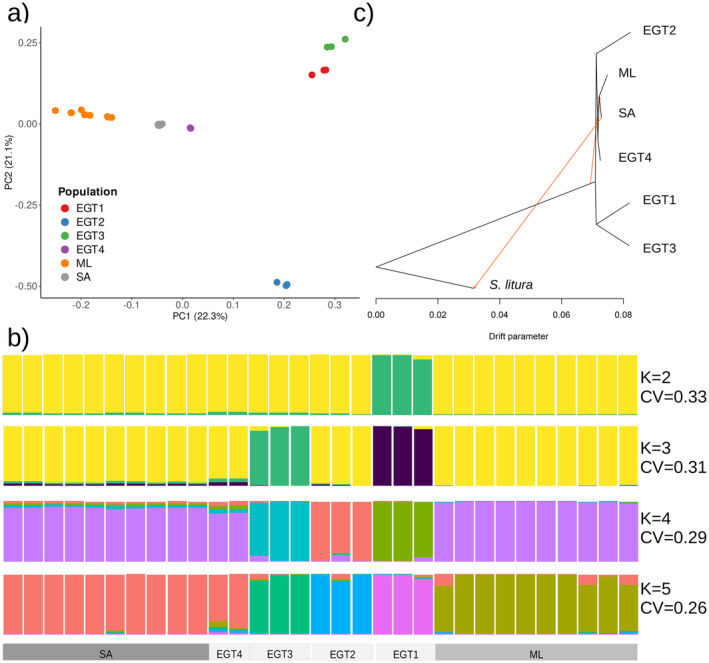
Genomic differentiation among groups (a) Principal component analysis results show genetic differentiation according to geographic origin where EGT are divided into four subgroups (b). The ancestry coefficient analysis shows different ancestry among SA, ML, and EGT subgroups. Cross‐entropy is indicated beside the plot (c) Phylogenetic tree resulting from the TreeMix analysis (rooted on *S*. *litura* ) and showing two migration events.

In total, 25,417,806 SNVs were identified from these samples after filtering. Principal component analysis was performed to test the existence of diverged populations in *S. littoralis* (Figure 2a). The first principal component, which accounted for 22.3% of the total variance, separated the samples into three groups according to geographic origin (ML, SA, and EGT). F_ST_ between the SA and EGT was 0.0341, 0.0534 between the ML and EGT, and 0.0294 between SA and ML. All of these F_ST_ values exceeded the random expectation with 100 replications, implying genomic differentiation with statistical significance (*p* < 0.01). The second principal component, which explained 21.1% of the total variance, separated the EGT into four sub‐groups, which we will refer to EGT1, EGT2, EGT3, and EGT4 hereafter. EGT1 comprised the samples from cotton and tomatoes in Damanhour. EGT2 and EGT3 were composed of samples collected from cotton in Faiyum and from cotton and maize in Kafr‐El‐Sheikh, respectively. EGT4 comprised two samples from cotton in Faiyum and Kafr‐El‐Sheikh. These results suggest that the populations were diverged according to geographic origins, while EGT showed higher haplotype diversities than SA or ML.

We performed the ancestry coefficient analysis to infer the genetic relatedness of the ancestry of each sample. Each of ML and SA appeared to have shared ancestry, while the subgroups of EGT showed distinct ancestry (EGT1—EGT3) (Figure 2b). The proximity of EGT4 to the SA group in the PCA, as well as its clustering with SA and ML in the phylogenetic tree, suggests a shared ancestry or gene flow between these populations. This result again suggests that the populations of the analyzed samples are genomically differentiated according to their geographic origins and that EGT has higher haplotype diversities than the other groups.

### 3.2 Phylogeographic Analysis

**FIGURE 3:**
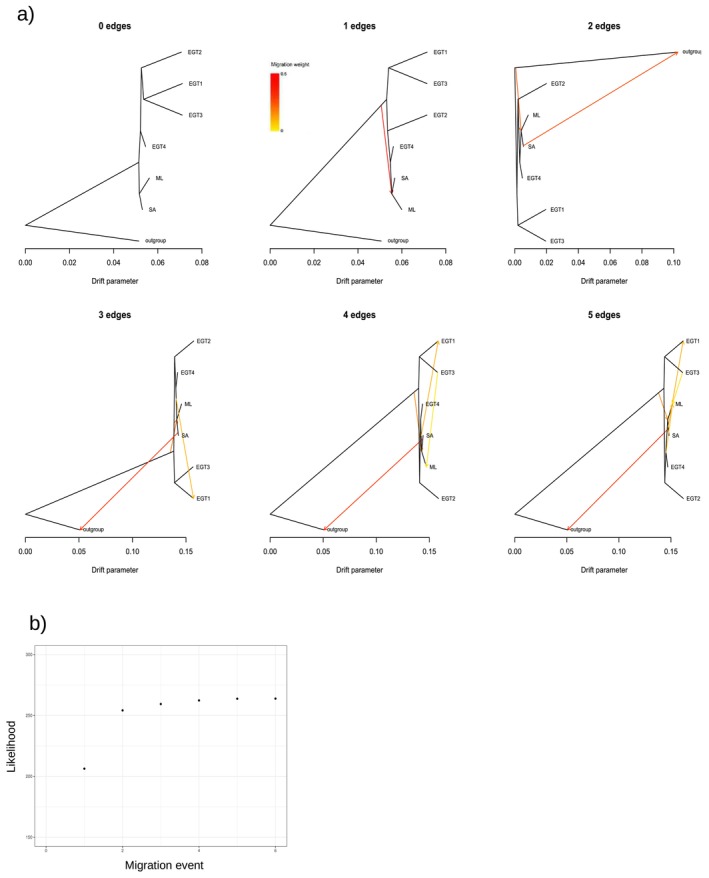
(a) Maximum‐likelihood phylogenetic trees with zero to five migration edges. The arrows indicate gene flow, and the colors show migration weight. (b) The plot shows the composite likelihood according to the number of migration edges.

We used the Treemix approach to infer the phylogenetic relationships among SA, ML, and the subgroups of EGT with the inference of migration among groups. With the inclusion of *S. litura* as an outgroup, we identified 2,476,369 SNVs. The resulting tree showed EGT positioned as the basal group in the phylogeny, with ML and SA identified as derived groups when the number of migration edges was assumed to be two (Figure 2c). Gene flow was detected from *S. litura* to the common ancestor of the ML and SA. When we assumed different numbers of migration edges between zero and five, a consistent phylogenetic pattern was observed (Figure 3).

### 3.3 Loci With Restricted Gene Flow

**FIGURE 4:**
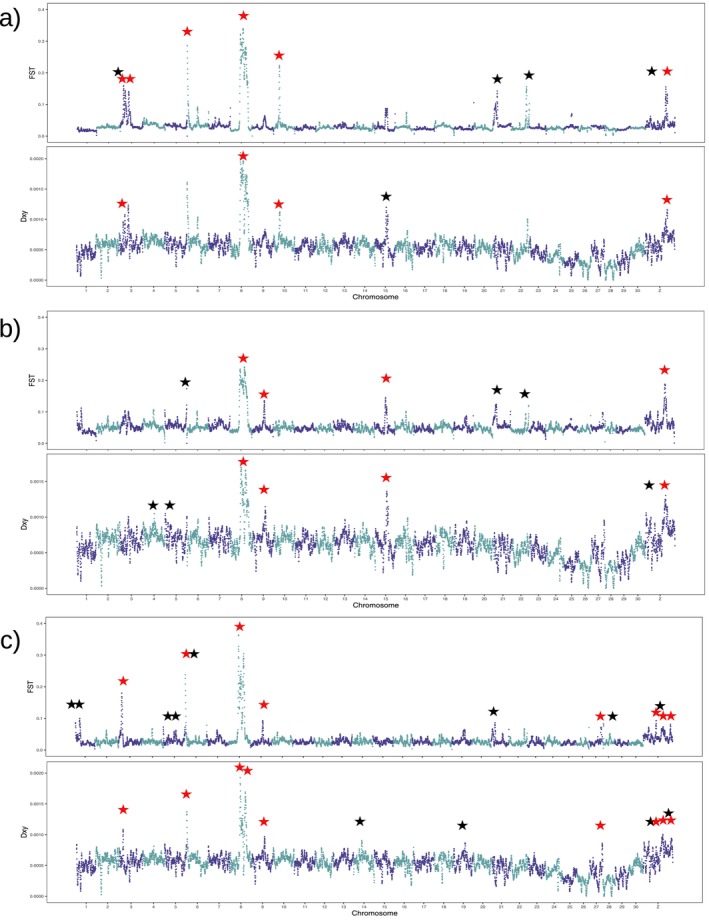
Genetic differentiation using F_ST_ and d_XY_ along chromosomes. The black asterisks show F_ST_ or d_XY_ outliers, while the red asterisks indicate overlapping F_ST_ and d_XY_ outliers (a) between the SA and EGT (b) between the ML and EGT, and (c) between ML and SA.

We tested the existence of genomic loci at which gene flow among the groups is restricted. For this purpose, first, F_ST_ was calculated along the chromosomes to identify OGD between groups with a 2% threshold (Figure 4). In total, 10, 7, and 16 outliers were identified between SA and EGT, between ML and EGT, and between ML and SA, respectively. As F_ST_ outliers could be generated solely from reduced intra‐group genetic diversity rather than restricted gene flow (Cruickshank and Hahn 2014), we also identified d_XY_ outliers with the same 2% threshold (Figure 4).

**FIGURE 5:**
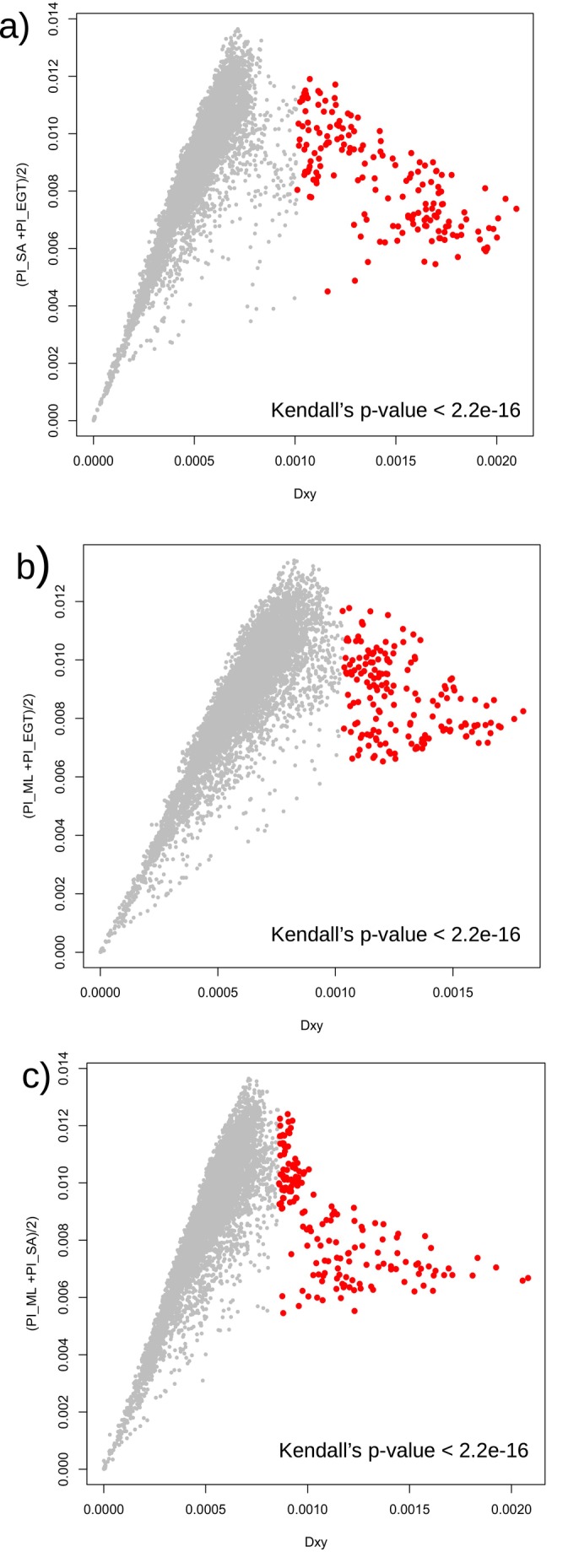
Relationships between d_XY_ and the averaged *π* of two populations between (a) SA and EGT, (b) ML and EGT, and (c) ML and SA. The red dots indicate the loci within OGD (outliers of genetic differentiation).

Seven, seven, and thirteen d_XY_ outliers were found between SA and EGT, ML and EGT, and between ML and SA, respectively. d_XY_ showed a strong positive correlation with π for each population (Kendall's Rank Correlation Test, *τ* = 0.689, 0.662, and 0.662 for SA‐EGT, ML‐EGT, and ML‐SA respectively, *p* < 2.2 × 10^−16^ for all comparisons) (Figure 5). However, the d_XY_ outliers did not display particularly high *π*, excluding the possibility of mutation hotspots. In total, six loci exhibited common outliers for F_ST_ and d_XY_ between SA and EGT, four loci between ML and EGT, and eight loci between ML and SA. These genetic loci are denoted as OGDs.

### 3.4 Divergent Selection

**FIGURE 6:**
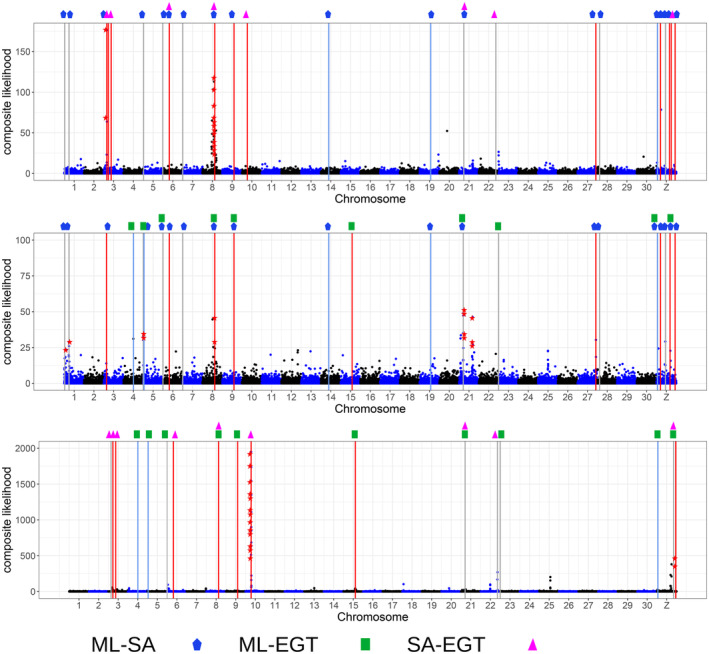
Composite likelihoods of selective sweeps are presented across the genome. Outliers of the composite likelihoods are indicated by the asterisks in (a) SA, (b) ML, and (c) EGT. Outliers for OGDs, F_ST_, or d_XY_ are marked with the vertical bars in red, gray, and blue, respectively. The observed comparisons (e.g., ML‐SA, ML‐EGT, and SA‐EGT) are indicated below the plots.

In total, 14, 13, and 15 composite likelihood outliers of selective sweeps were identified for SA, ML, and EGT, respectively (Figure 6). All composite likelihood outliers in SA and EGT were included in the OGDs. In ML, only two of the 13 composite likelihood outliers were included in the OGDs. Permutation tests showed that these associations have statistical significance (*p* < 0.001, < 0.001, and = 0.006 for SA, EGT, and ML, respectively), supporting the role of divergent selection in limiting gene flow at OGDs (Cruickshank and Hahn 2014).

**FIGURE 7:**
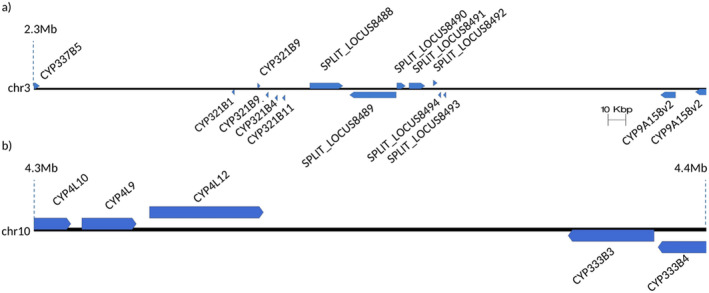
CYP cluster (a) for SA on chromosome 3 and (b) For EGT on chromosome 10.

**FIGURE 8:**
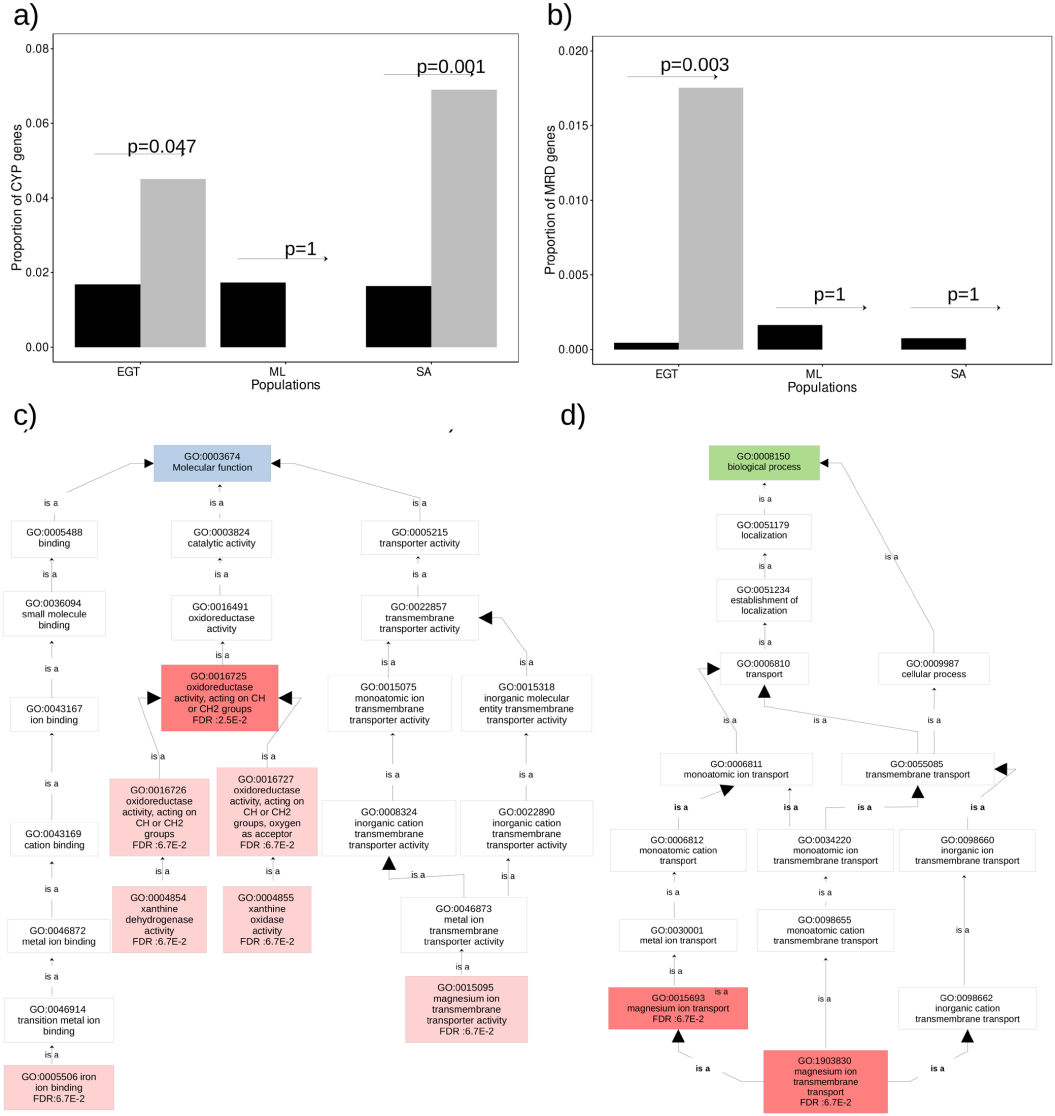
Enrichment Analysis (a) Bar plots show the proportion of CYP genes or (b) MDR genes within the OGDs targeted by selective sweeps (gray bars) and the proportion within the rest of the genomes (black bars). *p*‐values calculated by one‐tailed Fisher's exact test, are presented above the bar (c) Overrepresentation of molecular function category in SA (d) Overrepresentation of biological process category in SA.

In SA, the OGDs with outliers of the composite likelihood population contain a total of 124 genes, including 77 genes with known function (Appendix S1). These genes include eight CYP genes (CYP337B5, CYP321B1, CYP321B4, CYP321B11, two copies of CYP9A158v2, and two copies of CYP321B9) as a cluster on chromosome 3 (Figure 7). In EGT, 116 genes were identified including 57 genes with known function (Appendix S2). Again, these genes include five CYP genes (CYP4L9, CYP4L10, CYP4L12, CYP333B3, CYP333B4) located on chromosome 10 as a cluster (Figure 7). Two genes encoding multidrug resistance protein homolog 49‐like (MDR), and a gene encoding an odorant receptor (OR13) were also found in the Z chromosome. In ML, seven genes were identified from the targets of divergent selection including four genes with known function (Appendix S3). In SA and EGT, CYP genes are overrepresented in the targets (one‐tailed Fisher's exact test; *p* < 0.05) (Figure 8a). In EGT, MDR genes were significantly overrepresented in the targets (*p* < 0.05) (Figure 8b). The over‐representation of CYP or MDR was not observed from ML.

The GO analysis showed the overrepresentation of nine GO terms in the target of divergent selections within the OGDs of SA (Figure 8c,d). These GO terms were classified into three groups according to the hierarchical relationship. The first group is iron ion binding (GO:0005506) including eight CYP genes. The second group comprised five GO terms, including xanthine dehydrogenase activity (GO:0004854), xanthine oxidase activity (GO:0004855), oxidoreductase activity acting on CH or CH2 groups (GO:0016726), NAD or NADP as acceptor, (GO:0016727) oxidoreductase activity acting on CH or CH2 groups oxygen as acceptor, and oxidoreductase activity acting on CH or CH2 groups (GO:0016725). The overrepresentation of these GO terms involved two copies of xanthine dehydrogenase. The third group includes the molecular function of magnesium ion transmembrane transporter activity (GO:0015095), the biological processes of magnesium ion transmembrane transport (GO:1903830), and magnesium ion transport (GO:0015693). These GO terms are associated with two genes, the magnesium transporter nipa2 and the pre‐mRNA‐processing factor 40 homolog an isoform x2. ML and EGT did not show any overrepresented GO terms within the divergent selection at OGDs.

## 4 Discussion

In this study, we conducted population genomics analyses using 31 samples collected from southern Europe (ML), north Africa (EGT), and sub‐SA to test the existence of intraspecific population divergences and identify the evolutionary forces driving these divergences. Principal component analysis and ancestry coefficient analysis reveal clear grouping among SA, ML, and EGT, implying that the population divergence pattern is best explained by geographic origins (Figure 2a,b). Phylogenetic analyses show that EGT subgroups are basal phylogenetic groups, while the ML, SA, and EGT4 share a derived ancestral lineage (Figure 2c), suggesting the possibility that the analyzed ML, SA, and EGT4 samples were derived from a population including the EGT samples, which potentially represent a basal group of *S. littoralis* . We also observed a strong association between OGDs and the outliers of the composite likelihood of selective sweeps (Figure 6), supporting the role of natural divergent selection limiting gene flow among SA, ML, and EGT. It is worth noting, however, that the heterogeneous genomes of EGT samples might attenuate the footprints of selective sweeps, allowing only common selective sweeps across the entire EGT subgroup to be identified. Taken together, these results show in *S. littoralis* that natural divergent selection contributed to the divergence among geographic populations by limiting gene flow with the involvement of detoxification genes as selective targets. We, therefore, posit the possibility that, in *S. littoralis* , adaptive alleles in one geographic population are maladaptive in other populations and that this variation in adaptive fitness contributes to the genomic divergence among geographic populations by limiting gene flow. For example, the absence of detoxification genes targeted by selection in the ML population suggests that detoxification is likely not a major selective pressure in this population.

The genome scan to detect targets of divergent selections suggests that detoxification can be a major trait responsible for limited gene flow in OGDs. CYP gene families are overrepresented in the targets of divergent selection in SA and EGT (Figure 8a). The overrepresentation of the GO term “iron ion binding” was also closely linked to the eight CYP genes identified in SA. CYP genes are involved in the oxidation of a wide range of compounds, including insecticides, pheromones, and other foreign or endogenous substances, highlighting the importance of adaptation responses (Amezian et al. 2022; Feyereisen 1999; Giraudo et al. 2015; Liu et al. 2023). In SA, five CYP321B genes (CYP321B1, CYP321B4, CYP321B11, and two copies of CYP321B9) were included in the putative target of divergent selections within an OGS as a cluster (Figure 7). Previous studies highlighted the involvement of the CYP321B1 gene in detoxifying chlorpyrifos and β‐cypermethrin in *S. litura* (Wang et al. 2017). In *S. frugiperda* , the CYP321B1 gene is known to play a major role in the detoxification of chlorantraniliprole (Bai‐Zhong et al. 2020). The induction of CYP321B1 in the midgut and fat body of *S. litura* larvae, by exposure to tomato volatile substances (Luo et al. 2022), underlines its role in detoxification processes. This OGS also includes CYP genes belonging to the CYP9A family (two copies of the CYP9A158 gene), which is well known for its role in the detoxification of plant defense compounds and chemical insecticides, as observed in several species of *Spodoptera* (Amezian et al. 2022; Giraudo et al. 2015; Hilliou et al. 2021; Shi et al. 2023). In EGT, the target of divergent selection at an OGD includes five CYP genes belonging to CYP333B (CYP333B3 and CYP333B4) and CYP4L families, and (CYP4L9, CYP4L10, and CYP4L12), as a cluster (Figure 7). In *B. mori*, the CYP333 family is also suggested to be associated with xenobiotic metabolism (Ai et al. 2011). The CYP4L family is known to be involved in the detoxification of odorant or gustatory compounds (Amezian, Nauen, and Le Goff 2021; Li, Schuler, and Berenbaum 2007). CYP4L12 genes have a clear expression in the antenna and proboscis in *S. littoralis* , suggesting a potential specialization towards odorant or tastant substrates (Pottier et al. 2012).

The MDR genes are overrepresented in the targets of divergent selection in EGT (Figure 8b) and may play a role in insect adaptation to toxic environments. *H. armigera* has shown resistance to different classes of pesticides, with MDR being detected in resistant larvae, contrasting with their absence in pesticide‐susceptible larvae (Srinivas et al. 2004). Recent studies suggest that MDR activity is one of the features that protect herbivorous insects from toxins in their diet, by contributing to the elimination of defense compounds produced by plants (Aurade, Jayalakshmi, and Sreeramulu 2010; Sorensen and Dearing 2006).

The GO analysis suggests that detoxification involving xanthine dehydrogenase can also be associated with divergent selection in SA. Xanthine dehydrogenase caused the observed overrepresentation of the xanthine‐related GO terms at the targets of divergent selection within OGD. Xanthine is a xenobiotic molecule naturally synthesized in plants in the purine degradation pathway (Zrenner et al. 2006). In *S. frugiperda* , a species whose most common recent ancestor with *S. littoralis* originated ca. 12 million years ago (Kergoat et al. 2021), the enzyme Xanthine dehydrogenase contributes to the detoxification of xanthine by conversion to uric acid together with Xanthine oxidase (Slansky 1993). Hence, SA might have experienced adaptive evolution by unique selective pressures from plant xanthine. The functional implications of magnesium transport‐associated GO terms in the context of adaptation remain unclear.

Last, the odorant receptor OR13 was also identified as a target of divergent selection. This receptor is a pheromone receptor in *S. littoralis* , involved in the detection of a minor component of the sex pheromone blend in *S. littoralis* (de Fouchier et al. 2015). Although the sex pheromone blend composition of the collected samples has not been identified, it is known that *S. littoralis* females exhibit variations in this composition according to populations, especially in minor components (Quero et al. 1996). Thus, we speculate that variations in the sex pheromone composition led to variations in the corresponding receptors in males, as shown by selective sweeps specific to EGT in this study. As the differentiation of sex pheromones may cause prezygotic isolation in moths, slight variations in the pheromone communication channel may contribute to the observed population differentiation while the effect of allopatric distribution among EGT, SA, and ML should also be acknowledged.

We propose the possibility that the cultivation of different crop species or varieties, as well as insecticide use, may restrict gene flow due to divergent selection. The diversity of CYP genes in polyphagous insects is often attributed to adaptation to various plant xenobiotics (Cheng et al. 2017; Gouin et al. 2017), while this possibility remains to be tested (Dermauw, Van Leeuwen, and Feyereisen 2020). Moreover, in the absence of xenobiotics, resistance mechanisms may incur fitness costs (Smith et al. 2021; Tchouakui et al. 2020) (but see Ffrench‐Constant and Bass (2017)). If these scenarios hold, adaptive evolution through detoxification of one type of xenobiotic could be maladaptive in a population lacking exposure to the corresponding xenobiotic. For instance, CYP321B1 alleles adaptively evolved in SA for detoxifying a specific xenobiotic may impose fitness costs in EGT when this xenobiotic is absent, leading to the removal of this allele by purifying selection. Consequently, the gene flow of CYP321B1 between SA and EGT will be restricted. While speculative, we suggest that future studies should conduct detailed bioassays to test if this explanation is supported.

We also identified OGDs without the footprints of selective sweeps (Figure 6), indicating that non‐adaptive processes may contribute to gene flow restriction. One potential mechanism for this restriction is chromosomal rearrangement (Mackintosh et al. 2023). However, without a fitness advantage, such as in *Heliconius* species (Jay and Joron 2022), it is not easy to understand why chromosomal rearrangements have been maintained in a population despite their deleterious effects by reducing potential mating partners. Another evolutionary factor that could impede gene flow is genetic incompatibility. If the combination of derived loci introduced by gene flow and original loci leads to reduced fitness, genetic incompatibilities can arise and gene flow can be limited. In *Drosophila melanogaster* Meigen, widespread intraspecific genetic incompatibilities have been reported (Corbett‐Detig et al. 2013). Similarly, in *S. littoralis* , genetic incompatibilities could also be prevalent, especially among diverged geographic populations. Nonetheless, this non‐adaptive process of restricting gene flow might contribute to the onset of incipient speciation according to the Bateson‐Dobzhansky‐Muller model (Orr 1996).

Future studies could focus on four aspects. First, as mentioned above, different local environments that impose distinct adaptive pressures should be identified. We observed divergence in detoxification genes between EGT and SA, suggesting potential influences from the cultivation of different crop species or varieties, or variations in insecticide application. Second, the function of detoxification genes needs to be verified using functional genomics analyses, such as RNAi or CRISPR/Cas9. Intra‐species variation could be considered for this purpose. Third, conducting broader geographic sampling is essential to find a global pattern of population divergence in *S. littoralis* . Fourth, the cause of genomic divergence among EGT sub‐groups needs to be investigated with an increased sample size, as this divergence is likely driven by certain reproductive barriers induced by unidentified evolutionary forces.

In this study, we demonstrate that gene flow among geographic populations of *S. littoralis* is restricted by divergent selection acting mainly on detoxification genes. This result suggests that *S. littoralis* consists of populations with heterogeneous adaptive evolutionary histories. Specifically, the difference in evolutionary trajectories of detoxification genes implies that *S. littoralis* populations may possess differing capacities to penetrate plant defense systems and exhibit distinct levels of insecticide resistance. We argue that this heterogeneity should be taken into account in the development of adapted monitoring and management strategies. Furthermore, the meticulous investigation is essential to find the relationship between the evolution of detoxification genes and pest management activities.

## Author Contributions

**Karine Durand:** conceptualization (equal), formal analysis (lead), writing – original draft (lead). **Anne‐Laure Clamens:** data curation (supporting), writing – review and editing (supporting). **Bruno Le Ru:** resources (supporting), writing – review and editing (supporting). **Youssef Dewer:** resources (supporting), writing – review and editing (supporting). **Frédérique Hilliou:** formal analysis (supporting), writing – review and editing (supporting). **Camille Meslin:** data curation (supporting), resources (supporting). **Nicolas Nègre:** resources (supporting), writing – review and editing (supporting). **Gael J. Kergoat:** resources (supporting), writing – review and editing (supporting). **Emmanuelle Jacquin‐Joly:** data curation (supporting), resources (supporting), writing – review and editing (supporting). **Kiwoong Nam:** conceptualization (equal), supervision (lead), writing – original draft (supporting), writing – review and editing (supporting).

## Conflicts of Interest

The authors declare no conflicts of interest.

## Supporting information


Appendix S1.

## Data Availability

The resequencing data is available at NCBI SRA (PRJNA1118683). Computer programming scripts used in this study is available at https://github.com/karinedurand/Population_Genomic_Spodotera_littoralis/.
